# Whole transcriptome sequencing identifies BCOR internal tandem duplication as a common feature of clear cell sarcoma of the kidney

**DOI:** 10.18632/oncotarget.5882

**Published:** 2015-10-22

**Authors:** Annalisa Astolfi, Fraia Melchionda, Daniela Perotti, Maura Fois, Valentina Indio, Milena Urbini, Chiara Giusy Genovese, Paola Collini, Nunzio Salfi, Marilina Nantron, Paolo D'Angelo, Filippo Spreafico, Andrea Pession

**Affiliations:** ^1^ “Giorgio Prodi” Cancer Research Center, University of Bologna, Bologna, Italy; ^2^ Pediatric Hematology and Oncology Unit, S.Orsola-Malpighi Hospital, University of Bologna, Bologna, Italy; ^3^ Unit of Molecular Bases of Genetic Risk and Genetic Testing, Department of Preventive and Predictive Medicine, Fondazione IRCCS Istituto Nazionale dei Tumori, Milano, Italy; ^4^ Soft Tissue and Bone Pathology, Histopathology, and Pediatric Pathology Unit, Fondazione IRCCS Istituto Nazionale dei Tumori, Milano, Italy; ^5^ Pathology Unit, S.Orsola-Malpighi Hospital, University of Bologna, Bologna, Italy; ^6^ Department of Pediatric Hematology and Oncology, Istituto G. Gaslini, Genova, Italy; ^7^ Pediatric Hematology and Oncology Unit, A.R.N.A.S. Civico, Di Cristina and Benfratelli Hospital, Palermo, Italy; ^8^ Pediatric Oncology Unit, Department of Hematology and Pediatric Onco-Hematology, Fondazione IRCCS Istituto Nazionale dei Tumori, Milano, Italy

**Keywords:** CCSK, whole transcriptome sequencing, BCOR

## Abstract

**Purpose:**

Clear cell sarcoma of the kidney (CCSK) is a rare pediatric renal tumor that is frequently difficult to distinguish among other childhood renal tumors due to its histological heterogeneity. This work evaluates genetic abnormalities carried by a series of CCSK samples by whole transcriptome sequencing (WTS), to identify molecular biomarkers that could improve the diagnostic process.

**Methods:**

WTS was performed on tumor RNA from 8 patients with CCSK. Bioinformatic analysis, with implementation of a pipeline for detection of intragenic rearrangements, was executed. Sanger sequencing and gene expression were evaluated to validate BCOR internal tandem duplication (ITD).

**Results:**

WTS did not identify any shared SNVs, Ins/Del or fusion event. Conversely, analysis of intragenic rearrangements enabled the detection of a breakpoint within BCOR transcript recurrent in all samples. Three different in-frame ITD in exon15 of BCOR, were detected. The presence of the ITD was confirmed on tumor DNA and cDNA, and resulted in overexpression of BCOR.

**Conclusion:**

WTS coupled with specific bioinformatic analysis is able to detect rare genetic events, as intragenic rearrangements. ITD in the last exon of BCOR is recurrent in all CCSK samples analyzed, representing a valuable molecular marker to improve diagnosis of this rare childhood renal tumor.

## INTRODUCTION

Clear cell sarcoma of the kidney (CCSK) is a rare pediatric renal tumor that represents 3–5% of all childhood renal tumors, being the second most common malignant neoplasia of the kidney after Wilms tumor in the 0–14 age range [1]. Average age at onset is 36 months, with an incidence twice higher in males than in females [2–4]. Differently from Wilms tumor, CCSK does not appear to be associated with predisposing syndromes or to occur in individuals with germline genetic mutations [4].

Outcome was markedly affected by the improvement in chemo-radiotherapy protocols, with a current 5-year overall survival rate of 86% and a 5-year event-free survival rate of 78% [5]. Relapses occur in about 15% of the patients, with a 5-year event-free survival after relapse of 18%, and 5-year overall survival of 26% [6].

Histologically, CCSK has a morphology similar to other renal tumors and lacks proper immunohistochemical markers [1]. Additionally, genetic abnormalities characterizing the disease are lacking, with few reports on cytogenetic features that are generally not recurrent. The absence of distinguishing clinical, histological and genetic features is at the basis of the frequent misdiagnosis of this tumor type, that however requires different and intensified therapeutic protocols than other pediatric renal tumors. Only the *t*(10;17) involving the YWHAE-NUTM2 fusion has been recognized as a recurrent genetic event in CCSK, though identified in just 12% of cases [7]. Though not directly useful for molecular diagnosis, some common features of CCSK were identified, as the activation of AKT and SHH pathways [8], and the presence of a gene expression signature characteristic of an embryonal and primitive nephrogenic origin [9]. Only very recently new molecular features emerged, as the internal tandem duplication of BCOR [10], and the recurrent hypermethylation of TCF21 [11].

The BCOR (BCL6 corepressor) gene is located on chromosome Xp11.4 and encodes for an ubiquitously expressed nuclear protein involved in transcriptional regulation of BCL6 and of class I and II histone deacetylases [12]. Through these interactions, BCOR regulates expression of genes involved in early embryonic development, mesenchymal stem cell function, and hemopoiesis [13]. While germline *BCOR* mutations are responsible for the X-linked oculo-facio-cardio-dental (OFCD) syndrome, somatic alterations were detected in retinoblastoma, sarcoma and leukemia [12–15].

The aim of this study was to employ the most recent Next generation sequencing (NGS) techniques to identify shared genetic events that can be used as molecular markers of CCSK, thus aiding the diagnostic process and clinical management of patients.

## RESULTS

A series of 8 patients among 14 cases diagnosed with CCSK from 2003 to 2013, for whom fresh frozen tumor samples were available, were enrolled in the study. Age at diagnosis was 23.5 months on average (range 14–37 months), with a male to female ratio of 5:3. No patient carried genitourinary anomalies or other syndromic features. Two patients had metastatic disease at diagnosis; while 5/8 received primary surgery, the other three were first treated with neoadjuvant chemotherapy followed by surgery. All the patients were then subjected to adjuvant chemotherapy following surgery, according to AIEOP TW-2003 protocol.

All the samples were negative for the presence of YWHAE-NUTM2 fusion transcript from *t*(10;17) translocation (data not shown). Whole transcriptome massively parallel sequencing was performed on tumor RNA, reaching an average depth of 66X of the regions covered >1X. Chimeric transcript analysis did not identify any private or shared inter- or intra-chromosomal fusion event. All the samples showed numerous deleterious Single Nucleotide Variants (SNVs) or Insertion/Deletion (Ins/Del), though none was common between all the samples.

**Figure 1:**
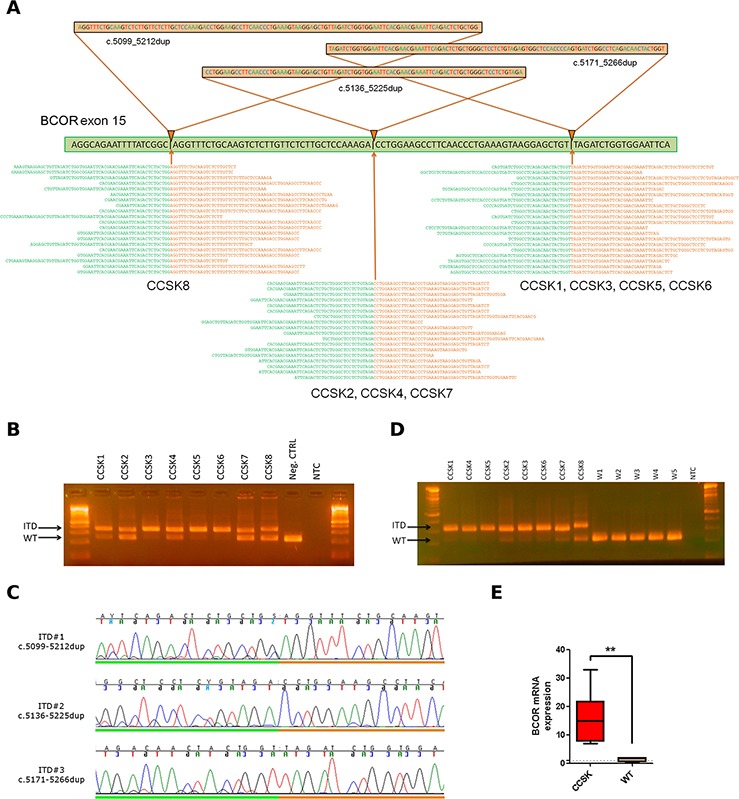
BCOR ITD detection by whole transcriptome sequencing **A.** Reconstruction of the three BCOR ITD events identified. Through *de novo* assembly and local realignment of unmapped reads, three different breakpoint regions were found, identifying three different ITD events in exon 15 of BCOR. The nucleotide sequences colored in green represents the wild type sequence while the ones in orange correspond to the duplicated segment. Reads overlapping the breakpoint regions are shown at the bottom. **B.** PCR amplification of BCOR exon 15 on tumor DNA of 8 CCSK and one negative control. Only CCSK samples carried the ITD (higher molecular weight). Two amplicons, corresponding to the duplicated and WT allele, were present in the 3 female patients. Two of the five male patients (CCSK1 and CCSK4) showed a fainter WT band, due to the presence of normal cells within the tumor tissue. Negative control carried only the WT allele (low weight). **C.** Chromatograms of the three different ITD breakpoint regions of the high weight bands obtained from amplification of CCSK tumor DNA. **D.** Amplification of BCOR from cDNA of 8 CCSK and 5 Wilms tumors (W1-W5). All CCSK expressed predominantly the ITD allele, while all Wilms tumors expressed only the WT allele. **E.** Evaluation of mRNA expression level of BCOR in the CCSK tumors with respect to the Wilms tumors, determined by quantitative RT-PCR. Expression level was normalized on GAPDH and significance (*P* = 0.004) was estimated with *t*-test statistic.

Analysis of intragenic rearrangements was executed by performing the local realignment of the highly expressed contigs previously assembled *de novo* from all the reads not mapping on the hg19 nor on the hg38 Reference Genome, including alternate sequences, haplotypes and ribosomal DNA. The BLASTN output was filtered excluding all the contigs having segments mapping on multiple genes, and the full-length contigs aligned entirely on a single transcript. This analysis enabled the detection of an average of 36 putative intragenic chimeras per patient among which a recurrent event (8/8) suggesting a breakpoint within the BCOR transcript (Table 1). In particular we highlighted the presence of an Internal Tandem Duplication (ITD) of the 3′ end of the last exon of BCOR. There were three different insertions and corresponding breakpoints in the 8 patients, a c.5171_5266dup resulting in a p.L1724_W1755dup, present in 4 patients, a c.5136_5225dup, leading to p.D1712_V1741dup, present in 3 patients, and lastly a c.5099_5212dup, p.L1737_G1738ins38, in one patient. These three different ITD led to the insertion of 96, 90 and 113 nucleotides, respectively, in the 3′ end of exon 15 of BCOR (Fig. 1A). All the duplications were in frame, and led to the insertion of 30–38 amino acids in the PUFD domain of the protein. The abnormal allele was highly expressed, with an average of 520 reads supporting the breakpoints.

**Table 1:** NGS identification of an internal tandem duplication in the BCOR gene

Sample	Contig Length	Conting start	Conting end	Transcript start	Transcript end	BLASTN E-val	ITD
**CCSK1**	67	1	30	5237	5266	1.00E-08	c.5171_5266dup
		29	67	5169	5207	5.00E-14	
**CCSK3**	54	1	17	5250	5266	1.00E-05	
		16	54	5169	5207	4.00E-14	
**CCSK5**	77	1	40	5227	5266	2.00E-14	
		39	77	5169	5207	6.00E-14	
**CCSK6**	67	1	30	5237	5266	1.00E-08	
		29	67	5169	5207	5.00E-14	
**CCSK2**	58	1	22	5204	5225	6.00E-04	c.5136_5225dup
		20	58	5133	5171	4.00E-14	
**CCSK4**	75	1	39	5187	5225	6.00E-14	
		37	75	5133	5171	6.00E-14	
**CCSK7**	69	1	33	5193	5225	2.00E-10	
		31	69	5133	5171	6.00E-14	
**CCSK8**	51	1	22	5191	5212	5.00E-04	c.5099_5212dup
		23	51	5099	5127	3.00E-08	

The analysis detected three different breakpoints on exon 15 of BCOR transcript (ENST00000378444). For each sample the BLASTN algorithm locally aligns the contigs in two different regions mapping on exon15 of BCOR: the first portion of the sequence overlaps a BCOR region closer to 3′-end with respect to the last portion, suggesting a duplication and insertion event.

The presence of this ITD event was confirmed by PCR followed by Sanger sequencing of the region of interest on tumor DNA. Amplicon size was checked on agarose gel thus confirming the dimension of the altered allele detected by NGS (Fig. 1B). Female patients carried both wild type and mutated allele. Sanger sequencing confirmed the exact breakpoint sequence of the three types of ITD detected (Fig. 1C). Amplification of the same region on cDNA, showed that at the mRNA level also female samples expressed predominantly the mutated allele, and that these ITD were not detected in Wilms tumors control group (Fig. 1D).

Gene expression analysis was evaluated with quantitative RT-PCR, compared to a control group of 5 Wilms tumors, showing that only CCSK were characterized by a consistent overexpression of BCOR (*P* = 0.004) (Fig. 1E). No difference was found in the level of expression of BCOR nor in the type of ITD in the two patients with metastatic disease.

## DISCUSSION

Proper identification of CCSK is fundamental to ensure correct treatment protocols, however diagnosis is hampered by morphological similarity to other renal tumors and lack of distinctive immunohistochemical markers [1].

Over the last decade, advances in NGS technology enabled simultaneous examination of numerous genes and many investigators have applied this technique with the aim of indentifying recurrent mutations or tumor driver genes in several types of cancer [16–18]. In this study we used NGS on a series of 8 CCSK for the discovery of molecular biomarker for this disease. Even though CCSK were shown to have a rather stable genome, as demonstrated by the absence of recurrent genetic changes identifiable by copy number analysis and whole genome sequencing, as performed by Gooskens et al [11], in our series it was possible to identify a recurrent ITD in the BCOR gene through the analysis of intragenic rearrangements. Three different types of in-frame tandem duplications were detected, all occurring in the last coding exon of BCOR. All the samples tested were positive for this type of alteration. This finding is consistent with a recently published analysis in which BCOR ITD was identified by RT-PCR in all the 20 CCSK tumors tested [10].

BCOR protein, namely BCL6 corepressor, was shown to specifically inhibit gene expression through its interaction with BCL6 and with specific class I and II of histone deacetylases [12]. The ITD identified in our work involved the PUFD domain of the protein, necessary for the epigenetic functions of BCOR, while maintaining the BCL6-binding domain. Thus, one possible explanation is that the ITD probably induces aberrant methylation and modification in gene expression profiles. Furthermore, BCOR was found highly expressed in CCSK, suggesting that this gene could represent a key factor in supporting tumor growth, through its interaction with BCL6 and through the altered epigenetic signaling. Thus, further studies are needed to assess the potential role of BCOR as a therapeutic target in this malignancy.

In conclusion, we demonstrated that NGS technology is able to detect complex mutational events, such as ITD or other intragenic rearrangements, as in this case the BCOR internal tandem duplication of CCSK. Internal or partial tandem duplications have been up to now very rarely investigated, even if they can play an important role also in other tumor types or diseases. Our study shows that whole transcriptome data can be successfully exploited to identify these types of genetic lesions.

We suggest the detection of the partial duplication inside exon 15 of BCOR as part of the diagnostic process of pediatric kidney tumors, representing a useful biomarker for the diagnosis of CCSK.

## MATERIALS AND METHODS

### Patients and tumor specimens

From 2003 to 2013, 14 pediatric patients were diagnosed with CCSK and treated according to Associazione Italiana Ematologia Oncologia Pediatrica (AIEOP) 2003 protocol. Diagnosis was made by histological evaluation of surgical specimens or biopsies, and confirmed through a centralized review along the guidelines of the protocol. Therapy of AIEOP TW-2003 protocol for CCSK included primary nephrectomy, unless the tumor was deemed unresectable by the local surgeon and oncologist. Surgery was followed by three-drug weekly chemotherapy with vincristine, dactinomycin, doxorubicin for 6 weeks followed by local and/or metastatic site radiotherapy. After this phase, chemotherapy consisted of alternate 3-weekly courses of carboplatin/etoposide and Ifosfamide/doxorubicin, for a total of 34 weeks. Fresh-frozen CCSK tumor tissue was collected for eight patients, and stored at the Fondazione IRCCS Istituto Nazionale Tumori of Milan. A quality control of the frozen material through an Hematoxylin-Eosin stained slide was performed before the storage of the specimen. Fresh-frozen tissues of 5 Wilms tumors were collected and used as control group for the molecular analysis. Informed consent for participating in the studies had been obtained from patients and parents before treatment, according to national law and regulations. Ethical approval was obtained from medical ethical committees. Clinical features of the patients enrolled in the study are summarized in Supplementary Table 1.

### Next generation sequencing

Total RNA was isolated from fresh frozen tumor tissues using the RNeasy spin-column method (Qiagen, Milan, Italy). RNA libraries were prepared from 500 ng total RNA in accordance with Illumina's TruSeq RNA Sample Prep v2 protocol (Illumina, San Diego, California). Libraries were quality checked and sized with Agilent DNA 7500 chips on the Bioanalyzer 2100 (Agilent Technologies, Taiwan), then quantified using Quant-IT picogreen assay (Life Technologies). Paired-end libraries were sequenced at 2 × 80 bp read length, on the HiScanSQ Illumina sequencer. An average of 80.5 million reads per sample were analyzed for each sample, corresponding to 6.5 Gb of sequences/sample.

### Bioinformatic analysis

After quality control, the short reads were processed and mapped on the human reference genome (including alternate loci and ribosomal DNA) by TopHat/BowTie pipeline. Variation calling was performed with SAMtools and SNVMix2, thus identifying all the point mutations, insertions and deletions present in the sample (SNV and Ins/Del). Variants present in dbSNP, 1000 Genomes and Exome Variant Server, with frequency greater than 1% were excluded. Intergenic chromosomal rearrangements were detected with several bioinformatic tools (DeFuse, ChimeraScan and FusionMap). To detect intragenic rearrangements, unmapped sequences were *de novo* assembled with ABYSS in single-end mode [19]. For each sample, a subsets of new contigs were selected to include contigs with average coverage value above the whole sample average coverage and with a length >50 nt. The selected contigs were locally aligned with BLASTN against the whole set of ENSEMBL coding sequences and filtered in order to highlight the intragenic chimeric transcripts including the Internal Tandem Duplication. Analysis pipeline to detect intragenic rearrangements is summarized in Supplementary Fig. 1.

### RT-PCR and sanger sequencing

RNA was reverse transcribed to cDNA using the Transcriptor First-Strand cDNA Synthesis Kit (Life Technologies) with oligo dT primers. Primers specific for the YWHAE-NUTM2 fusion transcript derived from *t*(10;17)(q22;p13), were taken from O'Meara et al., 2012 [7]. Amplification was performed for 40 cycles with FastStart Taq Polymerase (Roche) and electrophorectic bands were visualized on ethidium bromide stained gels. Validation of BCOR ITD was performed on tumor DNA and cDNA with primers located on exon 15, outside the duplicated region (Fw 5′-CCATTGCAGAGGCAGAATTTTA-3′ and Rev 5′-CTGTACATGGTGGGTCCAGCT-3′). PCR products were cutted and purified from gel and sequenced using the Big Dye Terminator v1.1 cycle sequencing kit (Life Technologies) on ABI Prism 3730.

Gene expression comparison between 8 CCSK cDNA and a control group of other renal tumors (5 Wilms tumor samples) was performed by quantitative RT-PCR, with primers located on exons 12 and 13 (Fw 5′-CTCTTATGGTGCTGACCCCACC-3′; Rev 5′-CCACTGGCGTCATCATCATTG-3′).

## SUPPLEMENTARY FIGURE AND TABLE


