# Supplementary material for: Whole transcriptome sequencing identifies BCOR internal tandem duplication as a common feature of clear cell sarcoma of the kidney
Source: Oncotarget. 2015 Oct 22;6(38):40934–9. doi: 10.18632/oncotarget.5882 (PMC4747379; doi:10.18632/oncotarget.5882)
Supplement: Supplementary file 1 [file oncotarget-06-40934-s001.pdf]

SUPPLEMENTARY TABLE AND FIGURE

Supplementary Table S1: Characteristics of patients enrolled in the study

Patient	Sex	Months at diagnosis	Kidney	Stage	Metastasis at diagnosis
CCSK1	M	37	Left	I	–
CCSK2	F	20	Left	III	–
CCSK3	M	27	Right	IV	Bone
CCSK4	M	14	Right	I	–
CCSK5	M	21	Left	IV	Lung
CCSK6	M	27	Left	I	–
CCSK7	F	17	Left	II	–
CCSK8	F	25	Right	II	–

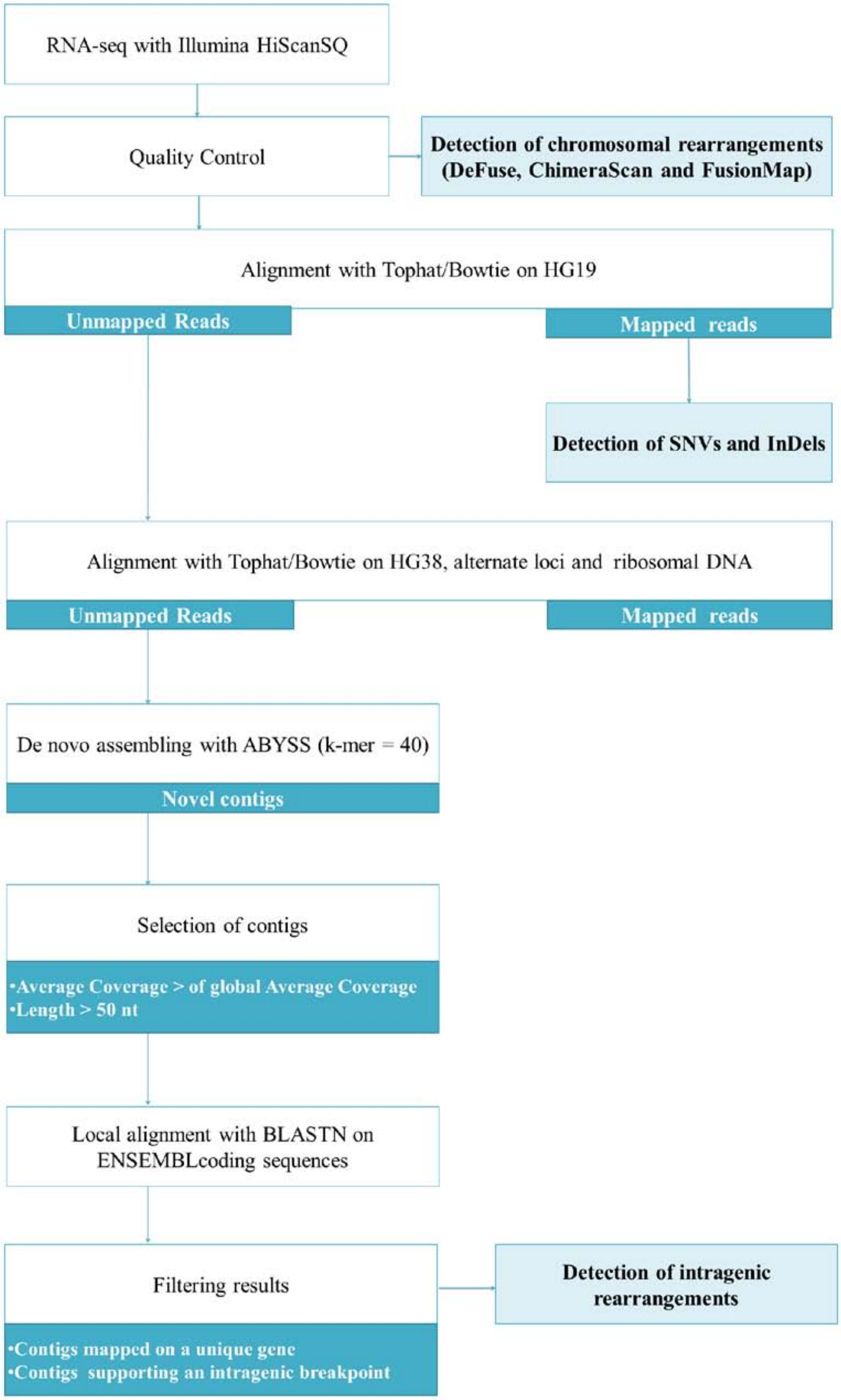

Supplementary Figure S1: Analysis pipeline to detect intragenic rearrangements.
